# Assessment and simulation of land use and land cover change impacts on the land surface temperature of Chaoyang District in Beijing, China

**DOI:** 10.7717/peerj.9115

**Published:** 2020-05-11

**Authors:** Muhammad Amir Siddique, Liu Dongyun, Pengli Li, Umair Rasool, Tauheed Ullah Khan, Tanzeel Javaid Aini Farooqi, Liwen Wang, Boqing Fan, Muhammad Awais Rasool

**Affiliations:** 1 School of Landscape Architecture, Beijing Forestry University, Beijing, China; 2 Department of Earth Sciences and Resources, China University of Geosciences, Beijing, China; 3 School of Ecology and Nature Conservation, Beijing Forestry University, Beijing, China; 4 Institute of Climate Change and Forestry Research, Beijing Forestry University, Beijing, China

**Keywords:** Land use and land cover change, Urban green vegetation, Land surface temperature, Urban dynamics, Urban planning, Markov model

## Abstract

Rapid urbanization is changing the existing patterns of land use land cover (LULC) globally, which is consequently increasing the land surface temperature (LST) in many regions. The present study is focused on estimating current and simulating future LULC and LST trends in the urban environment of Chaoyang District, Beijing. Past patterns of LULC and LST were identified through the maximum likelihood classification (MLC) method and multispectral Landsat satellite images during the 1990–2018 data period. The cellular automata (CA) and stochastic transition matrix of the Markov model were applied to simulate future (2025) LULC and LST changes, respectively, using their past patterns. The CA model was validated for the simulated and estimated LULC for 1990–2018, with an overall Kappa (K) value of 0.83, using validation modules in IDRISI software. Our results indicated that the cumulative changes in built-up to vegetation area were 74.61 km2 (16.08%) and 113.13 km2 (24.38%) from 1990 to 2018. The correlation coefficient of land use and land cover change (LULCC), including vegetation, water bodies and built-up area, had values of *r* =  − 0.155 (*p* > 0.005), −0.809 (*p* = 0.000), and 0.519 (*p* > 0.005), respectively. The results of future analysis revealed that there will be an estimated 164.92 km2 (−12%) decrease in vegetation area, while an expansion of approximately 283.04 km2 (6% change) will occur in built-up areas from 1990 to 2025. This decrease in vegetation cover and expansion of settlements would likely cause a rise of approximately ∼10.74 °C and ∼12.66 °C in future temperature, which would cause a rise in temperature (2025). The analyses could open an avenue regarding how to manage urban land cover patterns to enhance the resilience of cities to climate warming. This study provides scientific insights for environmental development and sustainability through efficient and effective urban planning and management in Beijing and will also help strengthen other research related to the UHI phenomenon in other parts of the world.

## Introduction

The relationship between land use and land cover change (LULCC) and land surface temperature (LST) is considered a popular issue in the researcher community in relation to environmental changes and sustainable development. Land use refers to anthropogenic deforestation, while land cover refers to the biophysical attributes of Earth’s surface in urban dynamics. As an essential part of worldwide sustainable development, urban cities have dramatically expanded in China, which is a matter requiring great attention (Tali et al., 2012; Zhang & Su, 2016). The association of urbanization and landscape patterns will offer support for urban ecological management (Sexton et al., 2013; Wang et al., 2019). Populations and socioeconomic activities developed in cities pose enormous sustainability challenges related to housing, infrastructure, food security, and natural resource management. Urbanization includes rapid increases in population, industrial structure, and landscape varieties (Zhang & Su, 2016). Large-scale LULCC was introduced in Beijing as a result of immediate urbanization from 1975 to 1997, which focused on developmental expansion and the erosion of intrusive agricultural land due to infrastructural changes in the concept of urbanization in the capital area (Zhang & Su, 2016; Zhang et al., 2018).

In recent years, scientists have recognized that LULCC evoked by human activities has immense impacts on the regional climate. Numerous studies have shown that urbanization will cause radical changes within the radioactive, thermodynamic, and hydrological processes at the land surface and thus modify local climatic changes in temperature, clouds, and precipitation (Carlson & Arthur, 2000; Huff & Changnon Jr, 1972). LST is an essential physical characteristic of the land surface that is directly influenced by LULCC, with implications for the study of climate change and related environmental impacts (Abreu-Harbich, Labaki & Matzarakis, 2014; Feng, Liu & Wu, 2014; Sobrino, Jiménez-Muñoz & Paolini, 2004; Walawender et al., 2014). In this case, the cover of vegetation and bare soil predisposed the partitioning of sensible, latent heat fluxes. Despite that, being a perilous physical property of the Earth’s surface, LST is challenging to measure over larger areas without the use of remote sensing. With the advent of thermal images acquired from satellites, it is now conceivable to monitor changes in LST over time and compare them with changes in LULCC (Amiri et al., 2009; Carlson & Arthur, 2000; Connors, Galletti & Chow, 2013; Ding & Shi, 2013). This complex relationship between land use cover types and environmental factors influences human livelihoods, LULCC detection, and mapping. That is, the relationship is relevant to many disciplines, including urban planning, climate change, and environmental monitoring (Carlson & Arthur, 2000; Ogashawara & Bastos, 2012; Rogan & Chen, 2004; Sexton et al., 2013; Weng, Liu & Lu, 2007). LULCC from one type to another, especially from farmland to metropolitan land/built-up areas, influences the process of energy exchange between the terrestrial surface and the atmosphere (Chen et al., 2006; Tonkaz & Çetin, 2007; Zhang et al., 2018). Characterizing the spatial heterogeneity of the urban heat island (UHI) as capable of changing with LULCC in urbanization is very significant for understanding ecosystem functions (Tali et al., 2012). Rapid urban growth has resulted in the final conversion of cultivated/agricultural land for construction uses, and this phenomenon is especially significant in black soil in China (Linda & Oluwatola, 2015). Various studies have indicated the same relationship between land use/cover change and civic thermal states.

The development of LULCC has become a dominant factor for environmental changes and managing resources. The over-expansion of anthropogenic activities leads to the symmetrical rise in planetary pollution. Several studies (Buyadi, Mohd & Misni, 2013; Chen et al., 2006; Tonkaz & Çetin, 2007) have considered the relative effects of LULCC on LST and have found congruous and convincing results. LULCC has become one of the substantial elements causing environmental vulnerability among  anthropological environmental systems (Verburg et al., 2004). LULCC modifies the spatial configuration of various land use varieties. It becomes necessary that we precisely identify LULCC at adequate scales with a significant time series. Thus, the higher perception of its impact on urban climate change improves the understanding of alternative environmental implications (Almazroui, Islam & Jones, 2013; Li et al., 2013). High rates of recent LULCC have been observed during urbanization in developing countries. It is especially relevant in China, where urbanization has become one of the most significant results of economic and social development (Chen et al., 2006; Ding & Shi, 2013; Oluseyi, Fanan & Magaji, 2009; Zhang & Su, 2016).

**Figure 1:**
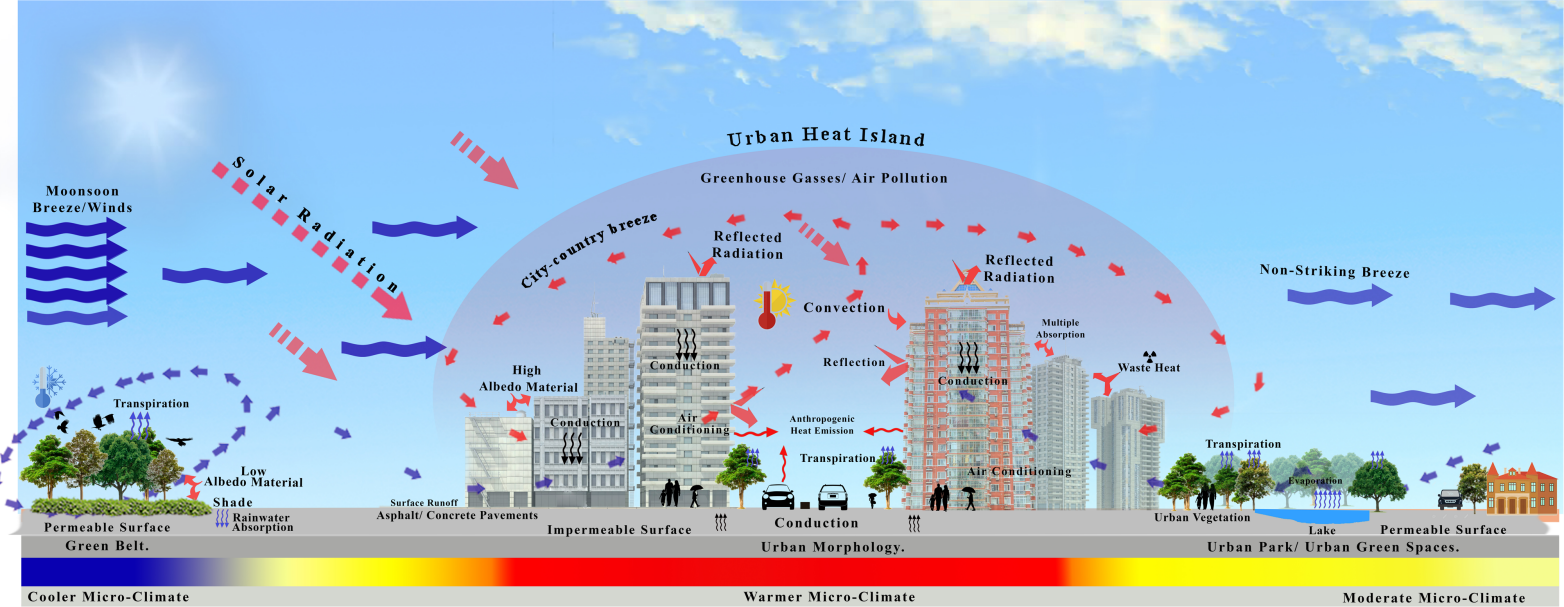
Conceptual framework for explaining the UHI phenomenon in city canyons.

Since the adoption of economic reform policies and the accelerated economic development in China, land use/cover has undergone tremendous changes. Expeditious urban growth has exerted enormous pressure on China’s environment (Pan & Zhao, 2005). China is the leading developing country in acreage and has been demonstrated the fastest urbanization in recent decades (Liu et al., 2014). Recent studies have shown the efficacy of large-scale urbanization on regional temperature variation in China (Weng, Liu & Lu, 2007; Weng, Lu & Schubring, 2004; Zhang & Su, 2016). There is proof that increasing global warming could be a result of anthropogenic activity during the past fifty years. The intensity of the UHI directly reflects the speed of urbanization, land use patterns, and building density (Fig. 1), illustrating the conceptual framework of how the UHI is structured and functions. The gradual increase in the surface structural changes of the city is divided into three layers depending on the climate and land use, as shown in Fig. 1. of the arriving monsoon winds entering from the eastern side of the city and are partially intercepted by the dense vegetative cover (green belt). This vegetation acts as a sieve to purify the air from heating and dust particles and enhances evapotranspiration, ultimately accelerating precipitation. After passing through the green belt, the air reaches the second layer where it is intercepted by built-up areas of the city, experiencing solar radiation interception and absorption phenomena, greenhouse gas emissions at the cost of anthropogenic disturbances, as well as building materials and engineering structures, making this microclimate warmer than the surroundings, termed an “urban heat island”. A part of the air crosses and moves towards the western side, which has a greater vegetative cover, more water bodies and less built-up area with a moderate microclimate.

This study aimed to quantify the effect of the urban land use patterns/transition of land use changes and land use changes on the surface temperature during 1990–2018 in the Chaoyang District of Beijing, China. We evaluated the relationship between LULCC and LST with changing climate. In this study, we hypothesized that climatic change might cancel out the effects and quantify the individual contributions of LULCC on LST in hotspot areas and its particular measures to mitigate their effects. The outcomes of this study provide scientific insight into urban heat island (UHI) issues and elucidate their causes and contributions to LULCC. This study also predicts and demonstrates future land use changes and LST scenarios in 2025 using the CA-Markov method.

### Study area, datasets and methodology

#### Study area

**Figure 2:**
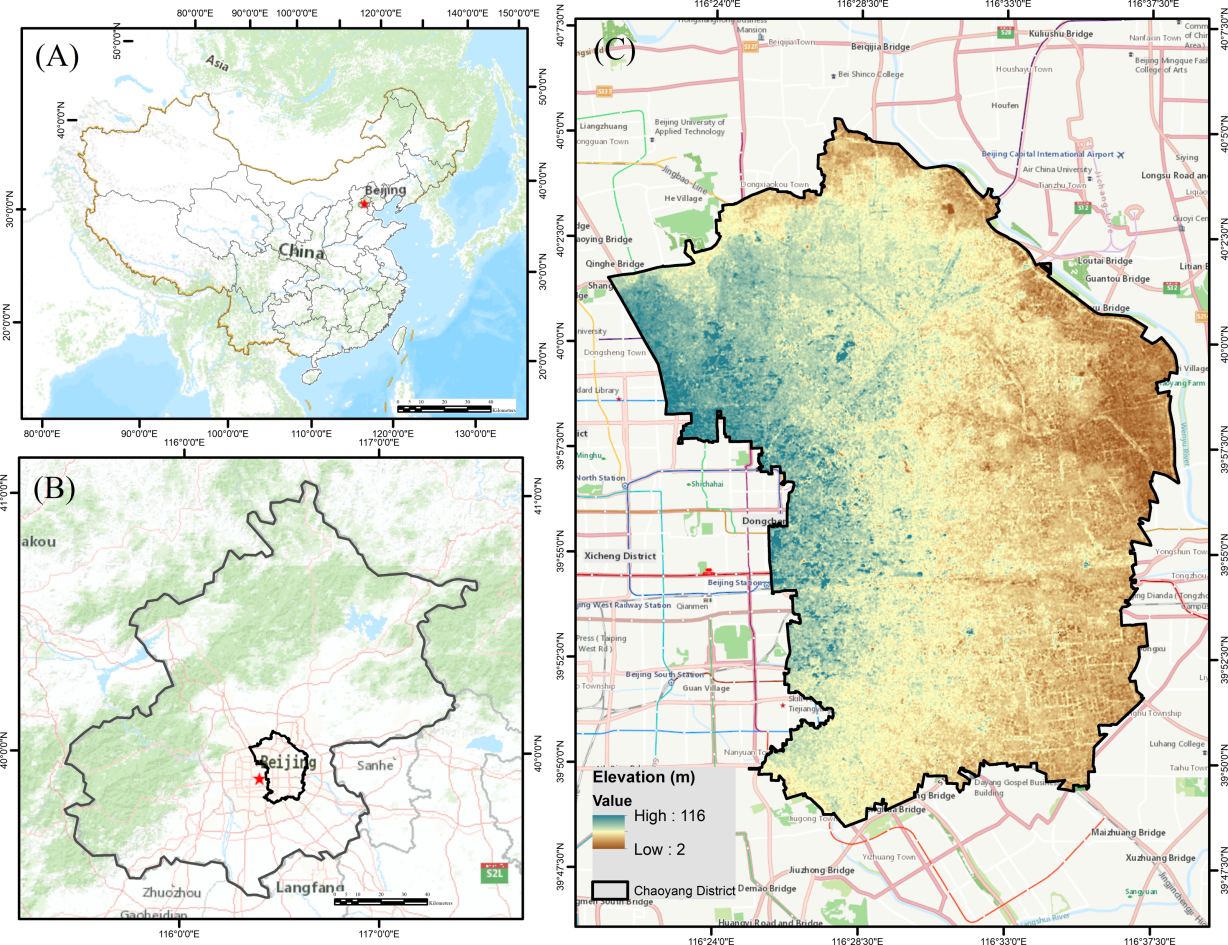
Map representing the geostrategic importance of the study area: (A) People’s Republic of China, (B) Beijing County, (C) Digital elevation model (DEM) of Chaoyang District showing elevation.

Chaoyang District is located in the middle of metropolitan Beijing (39°55′ N −116°26′E, ∼2 to ∼116 m) (Fig. 2). This district has an area of 478 km^2^ and a population of 3.5 million people between the 2nd and 5th ring roads. This district is the largest and most densely populated urban area of Beijing, with a density of 7,530 people/km^2^. The mean annual precipitation is approximately 644 mm, and the mean annual temperature is approximately 13.5 °C. Spring and autumn are short, and the average summer and winter period last approximately 3 and 5 months, respectively. The municipality, as well as the Chinese National Government, spends almost 0.5 million USD per day on the development of this district. The district has jurisdiction over 22 sub-district offices and 20 area offices.

**Figure 3:**
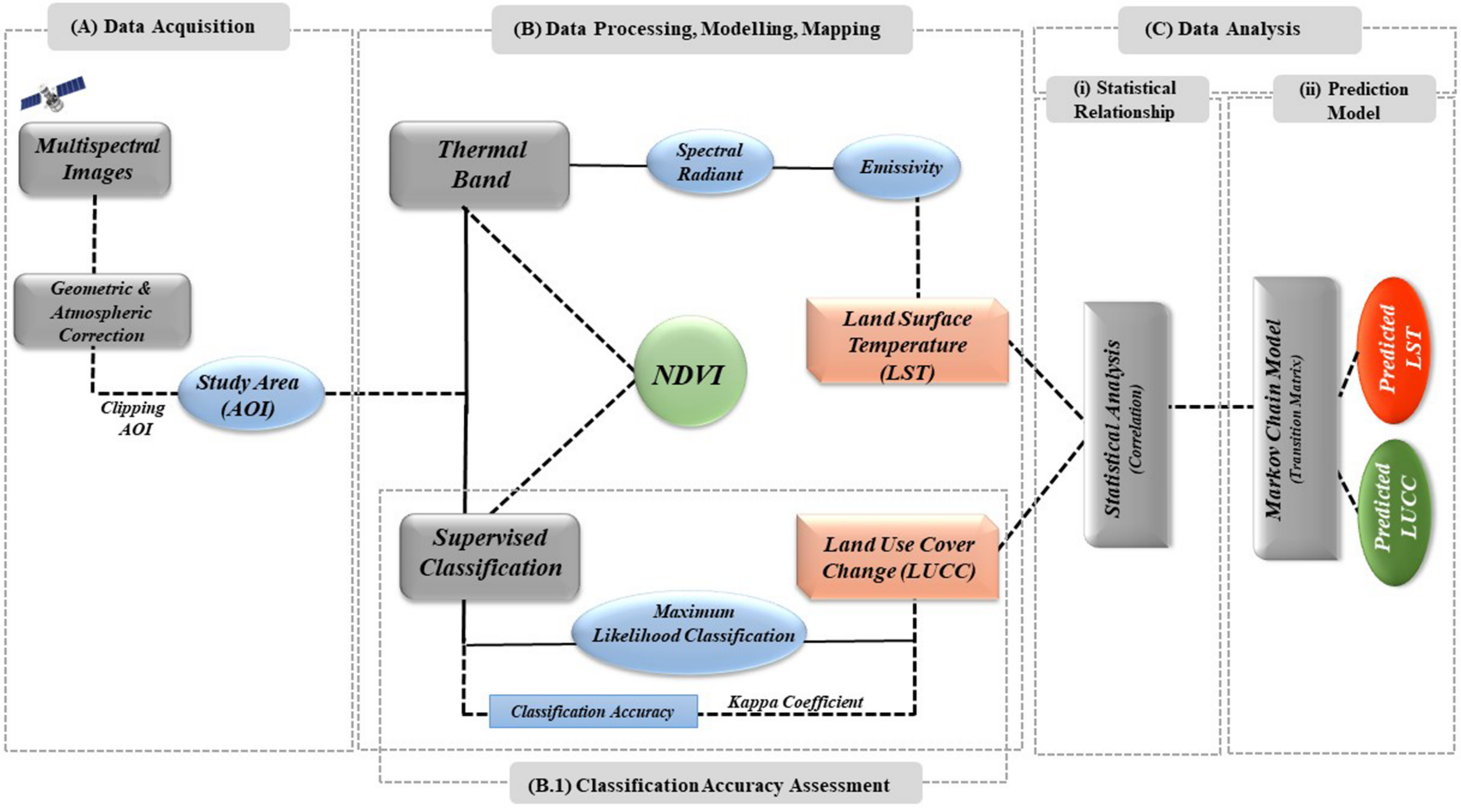
Methodology flow chart of the study.

### Materials and Methods

Spatial images of LTM (Landsat 4–5) and OLI (Landsat 8) with a 30 m resolution from 1990 to 2018 were obtained from the USGS Global Visualization Viewer (GloVis) and Earth Explorer. Each scene of the Landsat images was enhanced using the histogram equalization approach to attain a higher contrast of the images (Wu et al., 2004). According to the designed methodology (Fig. 3), these information sets were processed in ESRI ArcGIS version 10.5 to make a false colour composite (FCC). The study area was extracted from all spatial images by masking the georeferenced outline boundary map of Chaoyang. We calculated the normalized difference vegetation index (NDVI), land surface temperature (LST) and supervised classification method to ameliorate the classification results from Landsat images; additionally, we applied the Markov chain transition matrix to predict the future trends of the impact of LULCC on LST.

#### Data collection

The spatial images of LTM (Landsat 4–5) and OLI (Landsat 8) with a 30 m resolution from 1990 to 2018 were obtained from the USGS Earth Explorer for evaluating changes in LULC and LST. The entire Landsat scene cloud cover for the years 1990, 1997, 2004, 2011 and 2017 was approximately 3%–20%, but it was less than 1% in the study area (Table 1).

#### Computation of land use and land cover change (LULCC)

**Figure 4:**
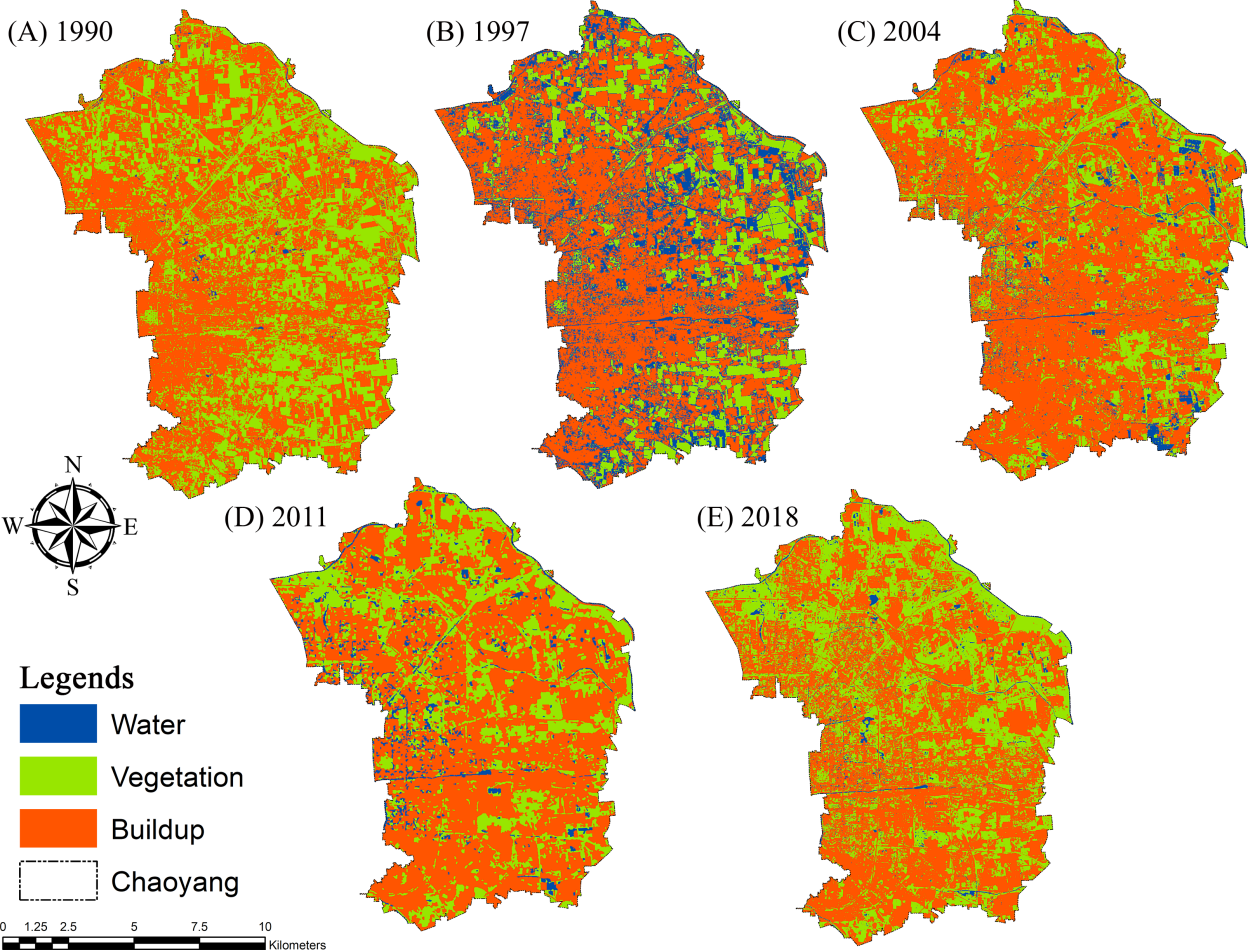
Land use and land cover change (LULCC) maps for (A) 1990, (B) 1997, (C) 2004, (D) 2011 and (E) 2018 in Chaoyang, Beijing.

Supervised classification is a method that is a “probability algorithmic program” applied within the ESRI ArcGIS 10.5 for land use/cover classification. Maximum likelihood classification (MLC) is a primer supervised classification scheme used in remote sensing tactics for data-image information. It has minimal computational time among alternative supervised tactics, in which the pixels that should not be unclassified become classified. Ground verification was performed in uncertain areas through supported bottom clothing where misclassified areas were corrected by imposition and rearranging the samples in ESRI ArcGIS version 10.5. The point of reference was meant to assess the mapping accuracy. The three basic land use/cover types identified within the study area were (1) vegetation, (2) developed/built-up area, and (3) waterbodies (Fig. 4).

**Table 1:** Details of the Landsat data used in this study.

Acquired date	Spacecraft ID	Sensor ID	Cloud cover
16 July 1990	Landsat-5	TM	∼16
21 July 1997	Landsat-5	TM	∼5
06 July 2004	Landsat-5	TM	∼6
26 July 2011	Landsat-5	TM	∼3
29 July 2018	Landsat-8	OLI_TIRS	∼19.13

#### Retrieval of land surface temperature (LST)

Radiometrically corrected Landsat images with the thermal infrared band (Band-6) were used to derive the land surface temperature (LST). The procedure, which involves the conversion of a digital number (DN) to an at-satellite brightness temperature, pursues correction for atmospheric absorption, re-emission and surface emissivity that has equally been utilized (Amanollahi et al., 2016; Li et al., 2016; Snyder et al., 1998; Sobrino & Jiménez-Muñoz, 2014; Sobrino, Jiménez-Muñoz & Paolini, 2004) to convert the spectral radiance to the top of atmosphere (TOA) brightness temperature beneath the hypothesis of uniform emissivity by these equations.

(1) \begin{eqnarray*}{T}_{k}= \frac{{K}_{2}}{\ln \nolimits \left( \frac{{K}_{1}}{{L}_{\lambda }} +1 \right) } \end{eqnarray*}

where *T*_*k*_ is the at-satellite brightness temperature in Kelvin (K), *L*_*λ*_ is the spectral radiance (W/m2*sr*µm), *K*_2_ is the calibration constant (K), and *K*_1_ is the calibration constant (W/m2*sr*µm).

We can calculate *L*_*sat*_ by using the following equation:

(2) \begin{eqnarray*}{L}_{sat}= \frac{ \left( {L}_{max}-{L}_{min} \right) }{QCA{L}_{max}-QCA{L}_{min}} \times \left( DN-QCA{L}_{min} \right) +{L}_{min}\end{eqnarray*}

where DN is the digital pixel number such as Band 6, *QCAL*_*max*_ = 255 is the maximum quantized calibrated pixel value corresponding to *L*_*max*_, *QCAL*_*min*_ = 0 is the minimum quantized calibrated pixel value corresponding to *L*_*min*_, *L*_*max*_ = 17.04 (mW/ *cm*^2^sr⋅ µm) is the spectral at-sensor radiance that is scaled to *QCAL*_*max*_, and *L*_*min*_ = 0 (mW/ *cm*^2^sr⋅ µm) is the spectral at-sensor radiance that is scaled to *QCAL*_*min*_.

From the calculation in Eq. (2), the radiance (*T*_*k*_)) is converted to surface temperature in Kelvin (K), which is then converted to Celsius (^o^C) by using the following equation:

(3) \begin{eqnarray*}{T}_{C}={T}_{k}-273.15\end{eqnarray*}

where *T*_*C*_ is the temperature in Celsius (°C), and *T*_*k*_ is the temperature in Kelvin (K).

**Figure 5:**
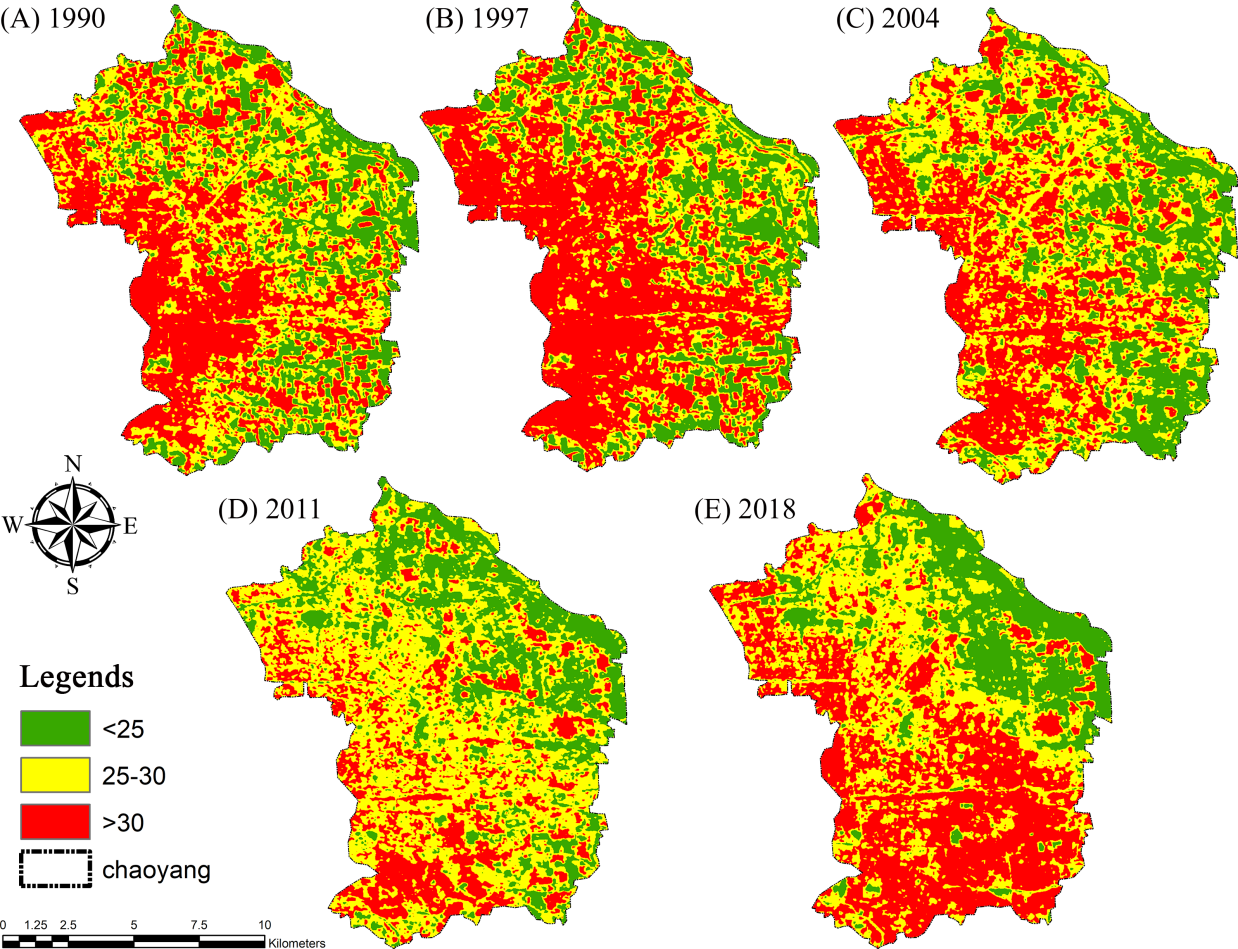
Land surface temperature (LST) maps for (A) 1990, (B) 1997, (C) 2004, (D) 2011 and (E) 2018 of Chaoyang, Beijing.

The land surface temperature (LST) was computed (Fig. 5) by using the following equation (Li et al., 2016; Sobrino, Jiménez-Muñoz & Paolini, 2004):

(4) \begin{eqnarray*}LST= \frac{{T}_{B}}{1+ \left( \frac{\lambda \cdot {T}_{B}}{\rho } \right) \cdot \ln \nolimits \left( \varepsilon \right) } \end{eqnarray*}

#### Markov chain model

This model is based on the creation of Markov stochastic process systems for the prediction of a status being changed to another (Muller & Middleton, 1994). The Markov chain model is usually used to simulate the changes, dimensions, and trends of land use cover changes. Additionally, the produced probability transition matrixes were used to forecast and determine the possible situations of future land-use change and urban growth patterns and to study the simulation trends of land surface temperature (LST) (Al-sharif & Pradhan, 2014). The prediction of future LULCC was performed by using a land-use change modeller (LCM) in Terrset (Clark Labs TerrSet 18.31), and land-use changes were analysed and the situation was projected to 2025 by CA-Markov. The LST can be calculated based on the conditional probability formula by using the following equations:

(5) \begin{eqnarray*}S \left( t+1 \right) & ={P}_{ij}\times S(t)\end{eqnarray*}

(6) \begin{eqnarray*}{P}_{ij}& = \left( \begin{array}{@{}c@{}} \displaystyle {P}_{11}\\ \displaystyle {P}_{21}\\ \displaystyle {P}_{n1} \end{array}\begin{array}{@{}c@{}} \displaystyle {P}_{12}\\ \displaystyle {P}_{22}\\ \displaystyle {P}_{n2} \end{array}\begin{array}{@{}c@{}} \displaystyle {P}_{1n}\\ \displaystyle {P}_{2n}\\ \displaystyle {P}_{n3} \end{array} \right) \end{eqnarray*}

Moreover,

(7) \begin{eqnarray*} \left( 0\leq {P}_{ij}\lt 1and\sum _{j=1}^{N}{P}_{ij}=1, \left( i,j=1,2,\ldots \ldots .n \right) \right) \end{eqnarray*}

where S(t) is the state of the system at time t; $S \left( t+1 \right) $ is the state of the system at time $ \left( t+1 \right) $; *P*_*ij*_ is the matrix of transition probability in a state.

## Results

### Land Use and Land Cover Changes (LULCC)

Land use cover changes (LULCCs) were computed for 1990, 1997, 2004, 2011 and 2018, focusing on vegetation, water bodies and the built-up area in the study area. The cumulative change calculated in vegetation was approximately 97.04 km^2^ (1990-2018), which was 90.18 km^2^ (1990) and 187.22 km^2^ (2018). An increase of 0.47 km^2^ was observed in water from 1990 to 2018. The built-up area was calculated to be 363.75 km^2^ in 1990 and 266.57 km2 in 2018, with an inverse accumulative change of 16% between 1990 and 2018 (Table 2).

**Table 2:** Distribution of the area (km^2^) according to land use cover classes during 1990–2018.

Year	Vegetation area (Km^2^)	Water area (Km^2^)	Built-up area (Km^2^)
1990	90.6462	10.233	363.5667
1997	104.8983	17.1747	342.3726
2004	118.449	24.1056	321.8913
2011	138.8997	21.5433	304.0029
2018	187.2189	10.7073	266.5197

**Notes.**

Km^2^ Square Kilometer

**Figure 6:**
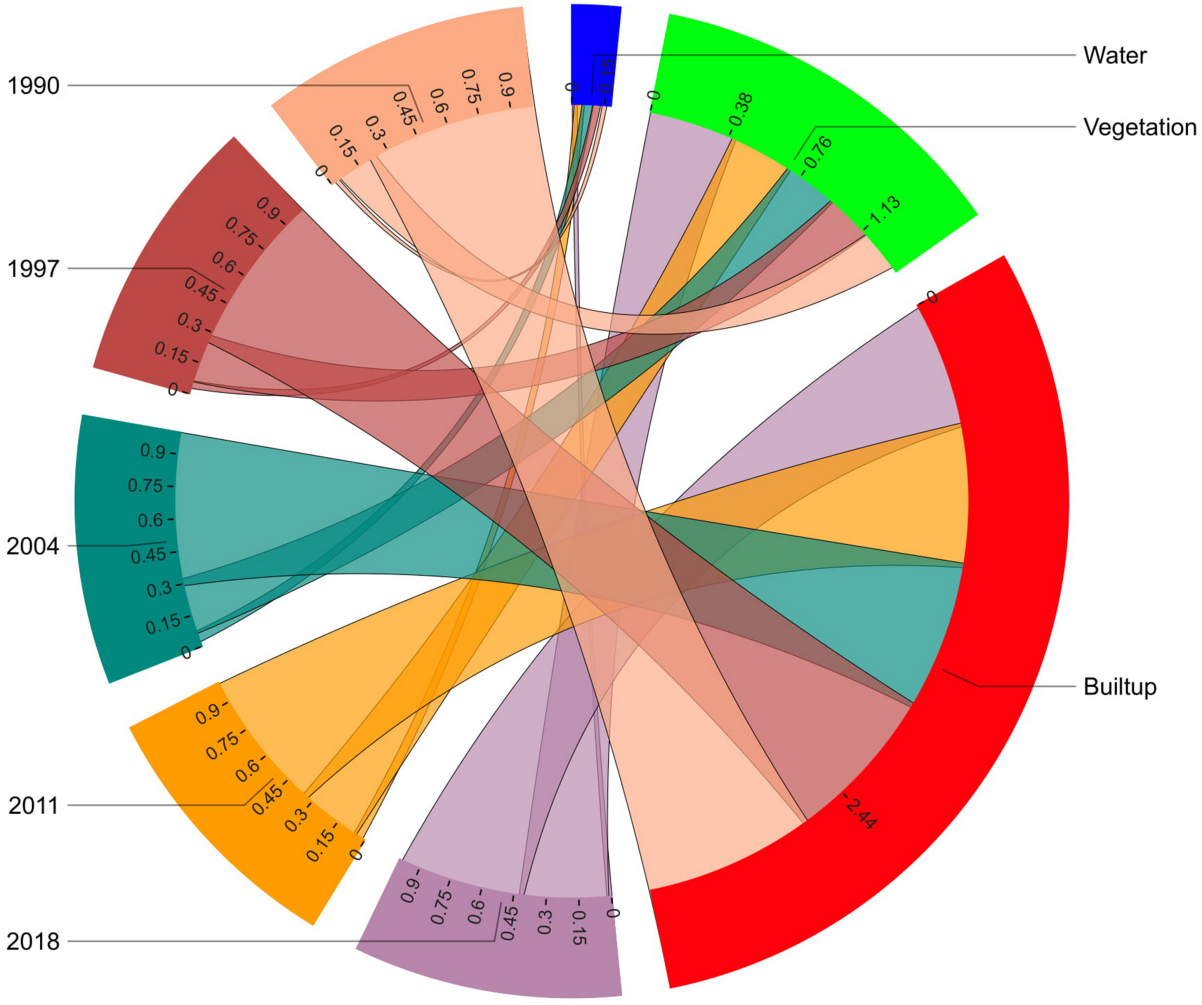
The chord diagram explicates the portion of land use land cover changes (LULCC) concerning the time series 1990–2018.

The chord wheel illustrates our results of the various land use categories for different years in proportion to others (Fig. 6). The vegetation area was calculated to be approximately 90.65 km^2^ in 1990, 104.89 km^2^ (1997), 118.45 km^2^ (2004), 138.89 km2 (2011) and 187.21 km2 (2018), with periodic increments of 16% from 1990-1997, 13% from 1997-2004, 17% from 2004-2011 and 35% from 2011-2018. A significant portion of the urban landscape was designated as grassland, shrubs, and ornamental plants.

The built-up area increased from 363.56 km^2^ in 1990, which was 78% of the area to 342.37 km^2^ (a 6% decrease) in 1997, and there were certain areas under construction that were calculated to be 18% (99.09 km^2^) and 29% (258.90 km^2^) of the built-up area in 1990 and 1997, respectively. From 2004-2018, a cumulative decrease of 12% was observed in the built-up area, as it was calculated to be 321.90 km^2^ in (2004), 304.01 km^2^ in 2011 and 266.52 km^2^ in 2018, with a 12% cumulative decrease. A cumulative decrease of 4% was observed in water bodies from 1990 to 2018. The water body areas calculated for 1990, 1997, 2004, 2011 and 2018 were 10.233 km^2^, 17.17 km^2^, 24.105 km^2^, 21.514 km^2^ and 10.70 km^2^, respectively.

**Figure 7:**
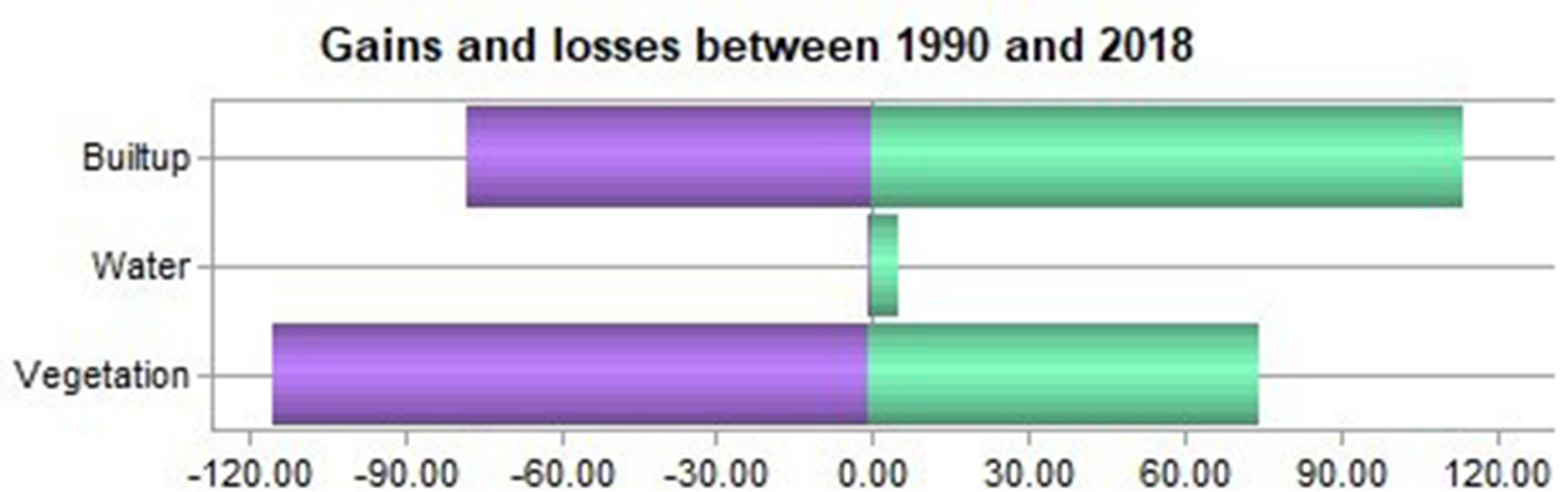
Proportional changes in LULCC in the study area between 1990 and 2018. Green bars represent the increment, and blue bars show the decrease in area (km^2^).

From 1990-2018, an area of 113.14 km^2^ experienced a 24% change in vegetation, and 0.35 km^2^ (∼0%) of water area was converted to built-up area. For the same period, a total of 74.61 km2 (16%) of built-up area and 0.22 km^2^ (∼0%) of water bodies changed to vegetation. Water bodies changed by approximately 3.14 km^2^ from built-up land and 1.92 km^2^ from vegetation, which was approximately 1% and ∼0% during this period, respectively (Fig. 7).

We observed a 17% loss, which was calculated to be approximately 77 km^2^, approximately 24% of the study area, 113.48 km2, had an increase, and 164.964 km^2^ (36%) remained the same during 1990-2018. However, for vegetation, 115.0632 km^2^ (25%) area had losses, 90.923 km^2^ (20%) was persistent and 74.830 km^2^ (16%) increased. Similarly, water bodies increased by 5.067 km^2^, an area of approximately 0.563 km^2^ decreased, and an area of 0.672km^2^ remained the same (Table 3).

**Table 3:** Statistics of the gain, loss and persistent area of the classes (1990–2018).

	Built-up Area		Vegetation Area		Water Area	
	Area	%age	Area	%age	Area	%age
Losses	77.7537	17%	115.0632	25%	0.5634	0%
Persistence	164.9646	36%	94.9293	20%	0.6723	0%
Gains	113.4828	24%	74.8305	16%	5.067	1%

#### Accuracy assessment of LULCC

The user’s accuracy of the supervised classification (LULCC maps) and the Kappa coefficient were determined by using terrset IDRISI. The overall classification accuracy was 94.26%, 92.96%, 91.86%, 89.56% and 91.89% for the years 1990, 1997, 2004, 2011 and 2018, respectively (Table 4). Although the overall Kappa coefficient was above 0.8399, it showed strong agreement (Cleve et al., 2008; Xiao et al., 2007).

**Table 4:** Accuracy computation of land use and land cover change (LULCC) maps between 1990 and 2018.

Year	User accuracy (%)	Producer accuracy (%)	Overall accuracy (%)	Kappa coefficient (%)
1990	96.37	86.48	94.26	0.91
1997	96.31	91.24	92.96	0.86
2004	93.54	93.65	91.86	0.87
2011	91.85	89.81	89.56	0.92
2018	94.78	92.74	91.89	0.89

### Estimation of Land Surface Temperature (LST)

The land surface temperature (LST) of Chaoyang District was calculated with minimum and maximum values of 26.45–50.9 °C in 1990, 26.45–53.49 °C (1997), 14.71–40.69 °C (2004), 19.28–39.54 °C (2011), and 13.22–44.32 °C (2018), respectively. The mean surface temperatures of the study area were 37 °C, 37.75 °C, 29.5 °C, and 31.50 °C in 1990, 1997, 2004, 2011 and 2018, respectively (Table 5).

**Table 5:** Computation of temperature (°C) for each time interval between 1990 and 2018.

Year	Min	Max	Range	Mean	STD
1990	26.45	50.99	24.54	36.47	3.01
1997	26.45	53.49	27.03	37.22	3.64
2004	14.71	40.69	25.97	28.67	2.51
2011	19.28	39.54	20.26	28.47	2.22
2018	13.22	44.32	31.10	35.08	2.71

**Figure 8:**
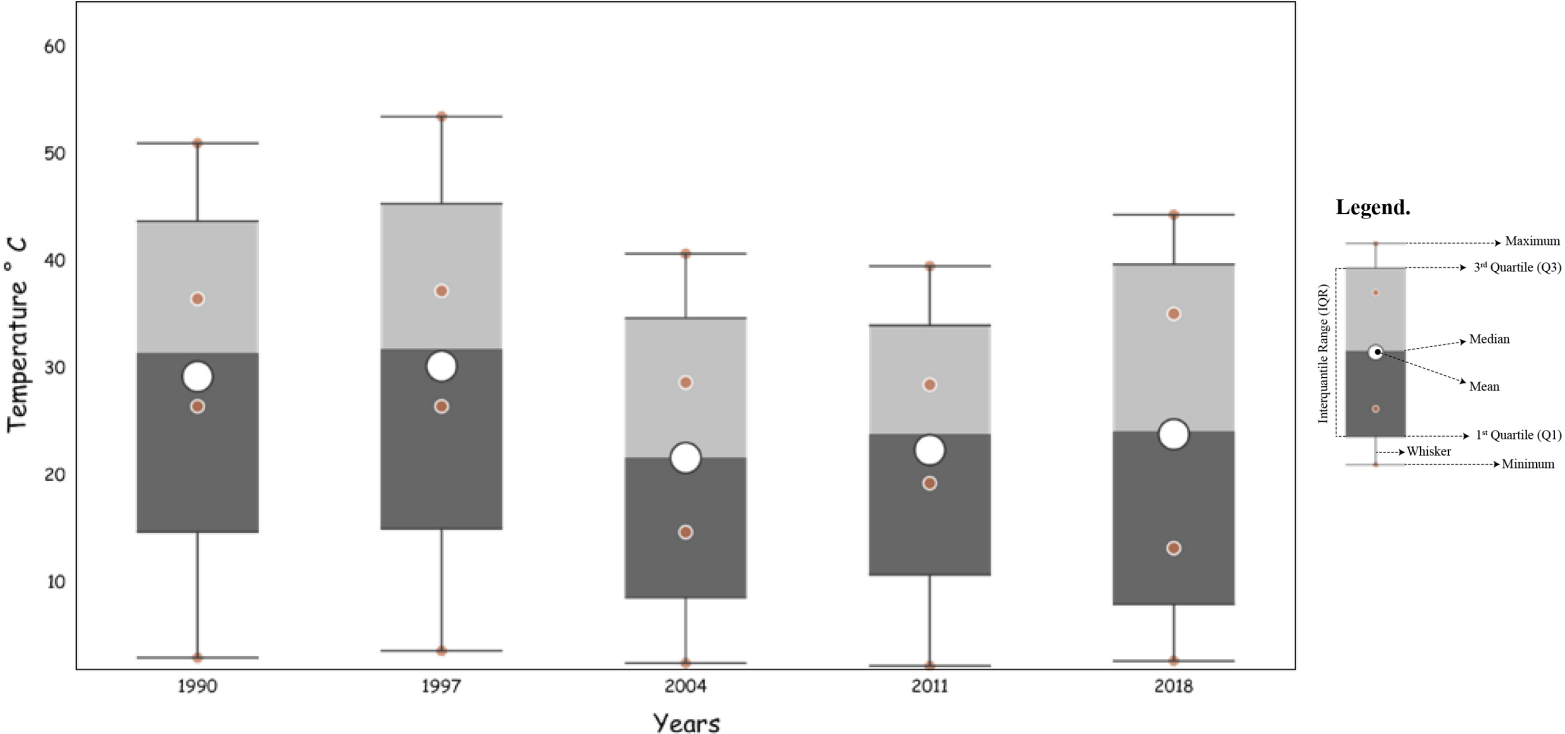
Land surface temperature (LST) during 1990–2018 in Chaoyang.

The results revealed that the ambivalence increased by approximately 10.35% in the LST. The following plot defines the minimum, maximum, mean and range values of land surface temperature in the study area during the respective period of 1990-2018 (Fig. 8).

From this study, the resulting mean surface temperatures in 1990, 1997, 2011, and 2018 were 37°C, 37.75°C, 29.5°C, and 31.50°C, respectively. The land cover types had various effects on land surface temperatures in between 1990-2018. It is apparent from this heat map that vegetation cover types had a considerable effect on land surface temperature during the study period, which indicates that this might be the result of transpiration, evaporation and absorption of other waste heat emissions (Tonkaz & Çetin, 2007; Wang, Zhan & Guo, 2016; Weng, Liu & Lu, 2007). Thus, the change in vegetation cover to different land cover types had given rise to an average increase of 5.5°C in radiant surface temperature during this period.

**Figure 9:**
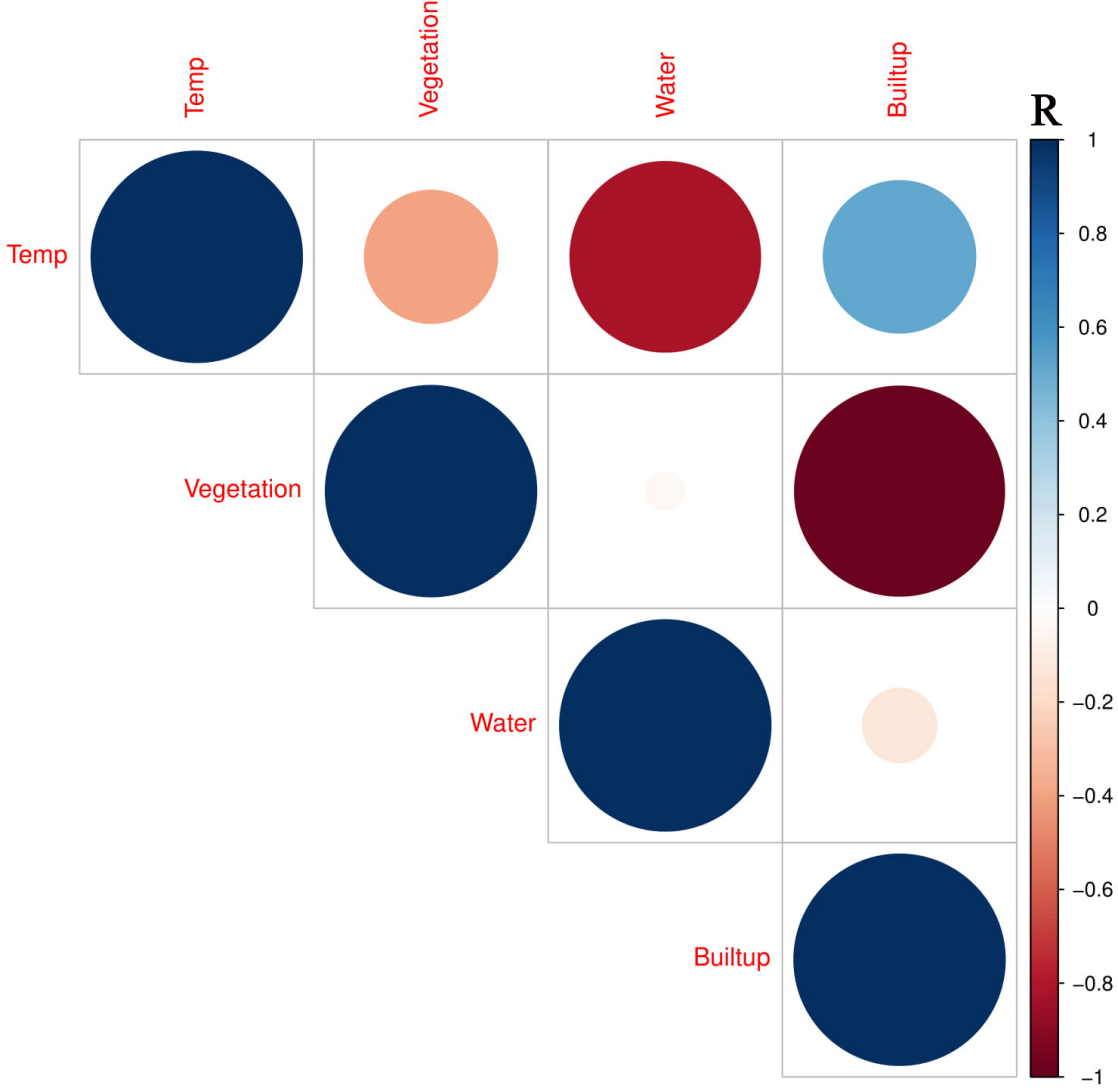
Corr-plot representing the linear correlation between the LULCC and mean LST for the period 1990–2018. The color bar represents the value of R.

### Relation of land use and land cover change on LST

A negative linear relationship between the vegetation and land surface temperature (LST) resulted in the value of *R* =  − 0.155, showing a non-significant relationship with *p* = 0.419. This corrplot highlights that vegetation index abates the LST in a particular area and time, and vice versa (Fig. 9). Many studies have supported this phenomenon: grasslands and ornamental plants have less impact on the reduction of LST than do forests and gardens (Chen et al., 2014; Fan et al., 2010; Wang, Zhan & Guo, 2016; Weng & Lo, 2001). Simultaneously, the water bodies strongly correlate negatively with land surface temperature by showing the resulting trend in the scatterplot very clearly. The R-value is −0.809, which shows its healthy significance level (*p* = 0.000), noting the impact of water bodies in the suburban area apart from a significant role in controlling the LST (Chen & Zhang, 2017; Duquène et al., 2009; Luo & Li, 2014). Urban water bodies shift the water content to moisten the mesosphere by evaporation. In contrast, built-up/developed areas or impervious surfaces and other dominant land cover types significantly contributed a large amount of heat flux to the overall phenomenon of urban heat islands (UHIs). A positive linear relationship exists between the land surface temperature (LST) and the built-up area (Fig. 9) (Chen & Zhang, 2017; Duquène et al., 2009; Wang, Zhan & Guo, 2016; Zhang & Su, 2016). These empirical estimates indicate a clear sense of the increment in LST when the plot shows an apparent positive correlation between the developed area and the LST, proving this relationship with an R-value of 0.526. The *p*-value authenticates the significance of this relation by showing the value of 0.003. These results established the phenomena that urban sprawl is the primary factor affecting the LST, which produces an abnormal heat flux in urban dynamics. This exchange of radiation/heat is considered a significant factor for the resulting UHI that contributes significantly to climate change in city canyons, as shown in Fig. 1. Therefore, LULCC does have an intense effect on the surface radiant temperature of a location, and it supports the phenomenon that anthropogenic LULCC is a main reason leading to an increase in LST in the urban micro-atmosphere (Jiang & Tian, 2010).

### Markov chain model analysis

**Figure 10:**
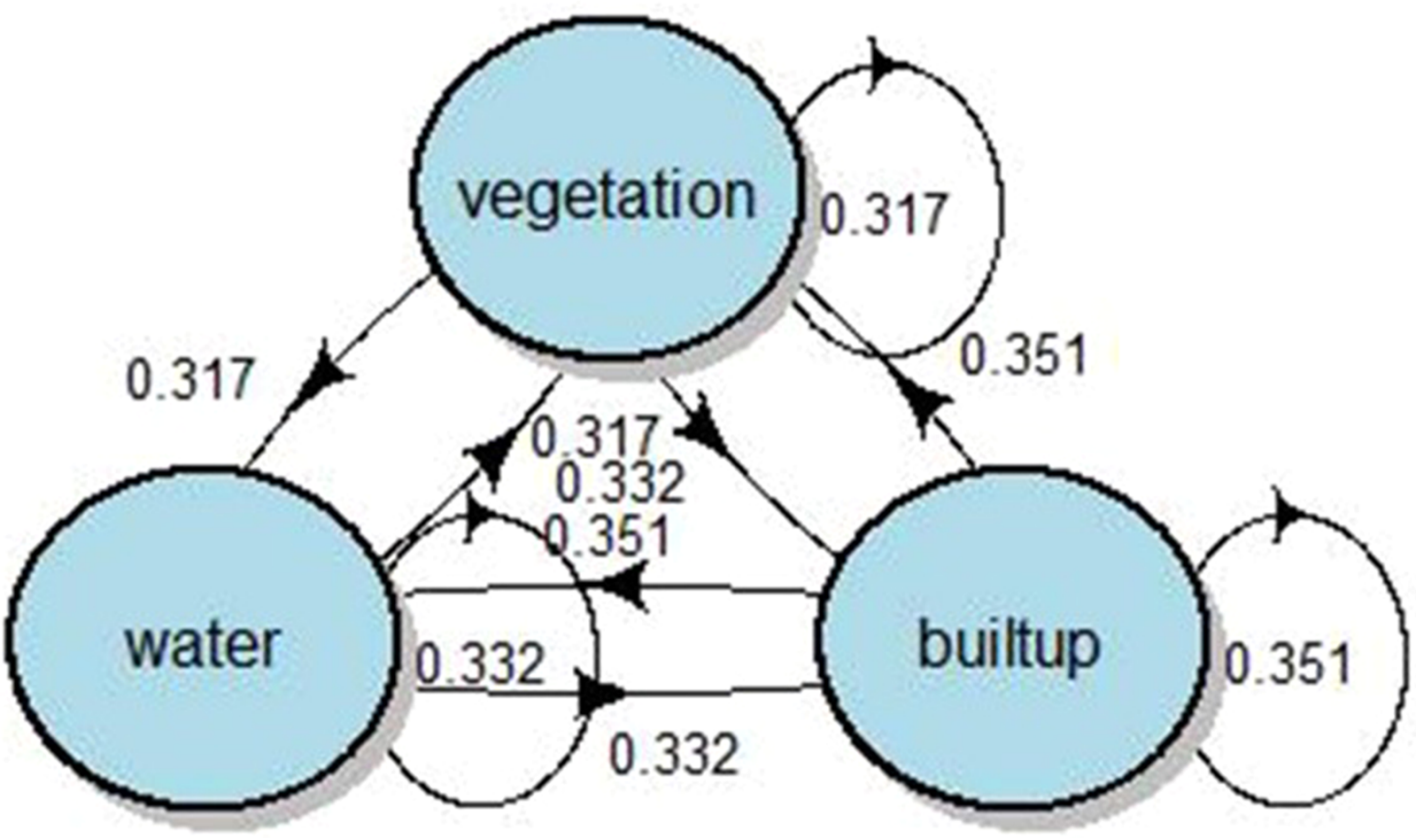
Markov chain’s Stochastic Transition Matrix structure of predictive analysis for LST-2025.

The Markov chain model was used to calculate the transition probability matrixes and the future potential percentages of land use and land cover change (LULCC) from projecting through data for the period of 1990–2018. The transition probabilities matrix of LULCC and LST in the period of 1990–2018 were plotted by generating the code script in the programming software RStudio (Fig. 10).

From the predictive model’s results, the LULCC in the study area will increase in the future; in this sense, the urban areas will increase to 178.83 km^2^; water bodies increase to 6.82 km^2^, and the increment in vegetation area is expected to be approximately 54.67 km^2^ in 2025 along with temperature increases of 10.74 °C, 10.70 °C and 12.66 °C in the vegetation, water and built-up areas, respectively (Table 6).

**Table 6:** Projected LULCC acreage (Km^2^) and its relative temperature (°C) during the period 1990–2025.

Year	Vegetation		Water Bodies		Built-up	
	Area (Km2)	Temp.(°C)	Area (Km2)	Temp.(°C)	Area (Km2)	Temp.(°C)
1990	90.18	32.66	10.23	28.27	363.57	37.50
1997	104.35	33.20	17.17	31.63	342.37	38.69
2004	118.45	27.04	24.11	25.85	321.89	29.36
2011	138.90	27.08	21.54	27.46	304.00	29.22
2018	187.22	33.87	10.71	32.26	266.52	36.07
2025^**^	164.92	44.61	5.98	42.98	283.04	48.73

**Notes.**

** Projected year

#### Simulation in LULCC and LST

**Figure 11:**
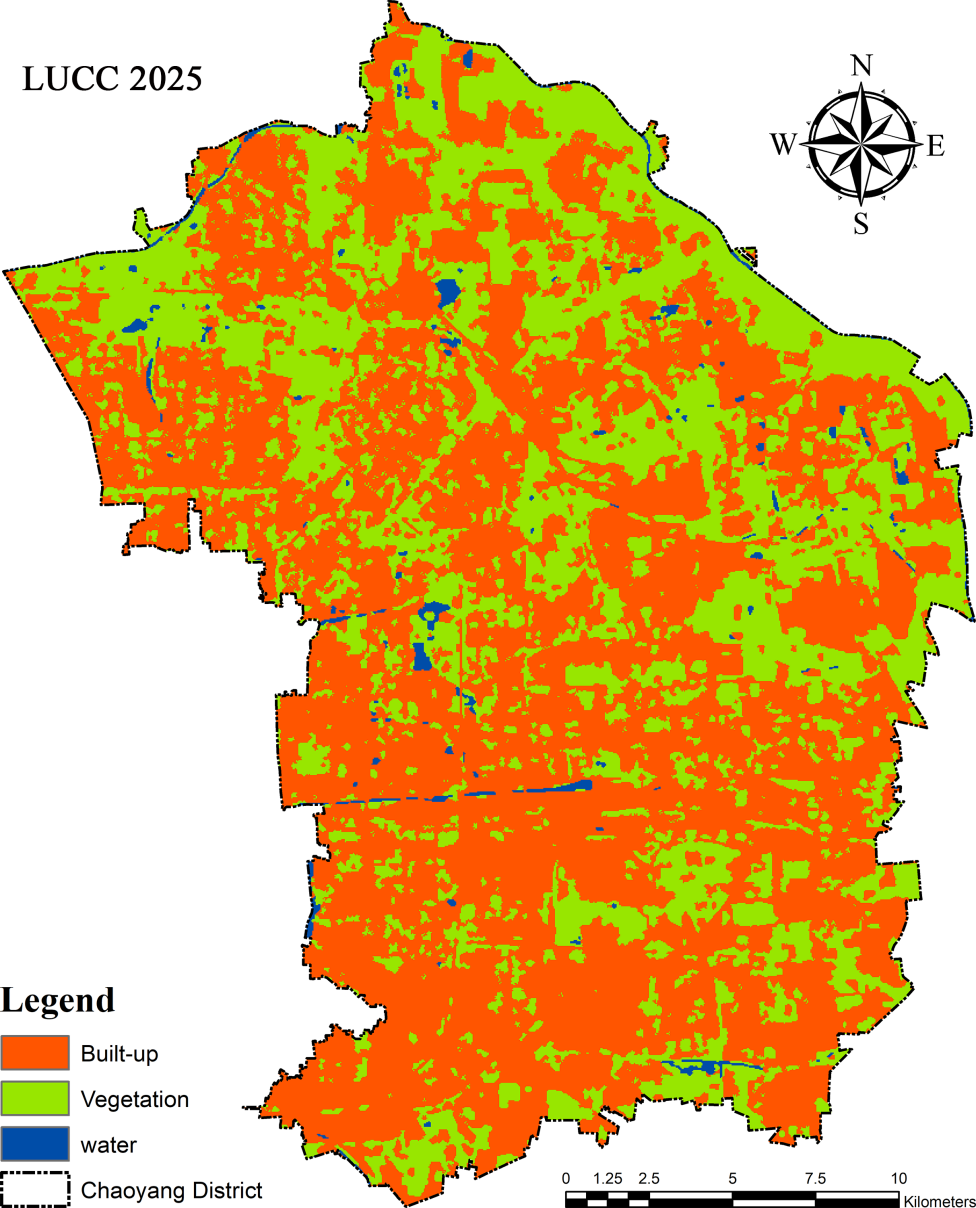
Projected map of land use land cover change (LULCC) for 2025 by CA-Markove.

The cellular automata (CA) and stochastic transition matrix of Markov chain models produced the LULCC and LST for the projected period of 2025. Approximately 12% of the negative change in green cover was estimated, which was approximately 164.92 km^2^, and the estimated temperature fluctuation will be 32%, with an approximately 10.74 °C rise in the vegetation area. The built-up area will expand by ∼6% (283.04 km^2^), adding an ∼35% (12.66 °C) rise in temperature for the projected period of 2025 (Fig. 11). Water bodies will decrease to 5.98 km^2,^ which is approximately −79%. The accuracy of the maps of projected land use cover change in 2025 was classified by the Kappa coefficient value, which was above 0.97.

## Discussion

### Quantitative relationship between LULCC and LST

**Figure 12:**
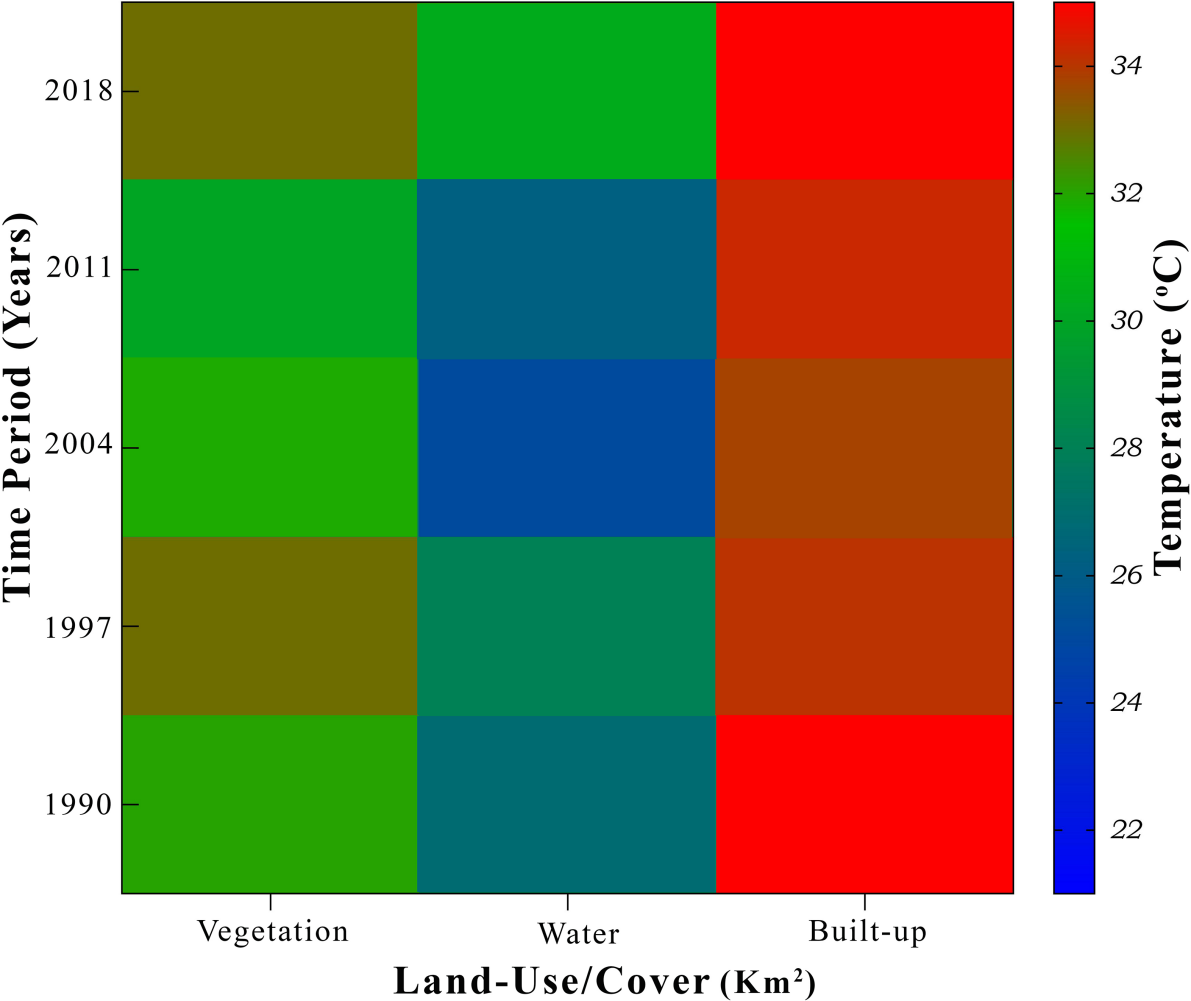
Relative temperature of various land use and landcover change (LULCC), during the study period 1990-2018 mentioned on axis. The color bar represents the mean temperature value of each segment.

Although the relationships between land surface temperature (LST) and land use and land cover change (LULCC) have been studied previously (Civco, 1993; Li et al., 2016; Ullah et al., 2019). In this study, remote sensing application was used to estimate temperature and land cover change, and study their relationship effects between 1990 and 2018. Moreover, we simulated these parameters for 2025 (Sejati, Buchori & Rudiarto, 2019; Ullah et al., 2019; Zhang & Su, 2016). Our results provide insights into testable hypotheses; urban climatic change quantifies the individual contributions of LULCC to LST in hotspot areas and its particular measures to mitigate consequential effects. Our findings showed that urban sprawl is the primary factor affecting land surface temperature (LST), which in turn produces an abnormal heat flux. This exchange of radiation/heat is considered a significant factor for the rise in the urban heat island (UHI), which contributes significantly to climate change (Pal & Ziaul, 2017; Zhang & Su, 2016; Zhang et al., 2009). LULCC has a relative impact on LST (Fig. 12), especially in urban areas (Tali et al., 2012; Wang, Zhan & Guo, 2016; Weng, Liu & Lu, 2007). Active management and understanding of LULCC is essential in the context of anthropogenic climate change and global warming (Morabito et al., 2016; Turner, Lambin & Reenberg, 2007; Ullah et al., 2019).

Land use and land cover change (LULCC) has a relative impact on land surface temperature (LST), especially in urban areas. Active management of LULCC and its understanding are essential in the context of anthropogenic climate change and global warming (Turner, Lambin & Reenberg, 2007; Wang, Zhan & Guo, 2016). The UHI effect is a result of various obvious reasons, such as macro/mesoscale climate, urban morphology, population density, geographic location, anthropogenic changes in biophysical features of the surface area/land use, wind corridor, population pressures and human lifestyle, and energy cycle. The observed trend in land use change reveals that the rate of land-use changes during the period from 1990 to 2004 was enormous and indicative of the need for rapid development of AOI at the time. This period was characterized by intense deforestation and demolishing of cropland for various developmental projects and shifted the cultivated land/agricultural land into the evident level of increase in barren land and built-up areas for housing and industry, characterized by a concomitant decline in total vegetation cover (VC) (Cai, Du & Xue, 2011; Chen et al., 2017). This rapid depletion of VC (high and low vegetation) had a wide range of impacts on the reduction of natural cooling effects because of the shading and evapotranspiration of plants and shrubs (Macarof & Statescu, 2017; Oluseyi, Fanan & Magaji, 2009). To buttress this fact, (Wang, Zhan & Guo, 2016; Weng, Lu & Schubring, 2004; Zhang et al., 2017) when we correlated the NDVI and LST, it showed the negative bonding between them. This result shows that green areas/VC act as a sink within an urban heat island (UHI) because of their cooling effects in an urban area. Our results in Fig. 11 clearly show that land cover has a very dominant impact on LST in the urban environment. This heat map explains the impact values of green space, blue space and water bodies on their LSTs according to their proportional area. Second, vegetation plays an important role in mitigating or controlling the temperature in urban areas (Weng, Lu & Schubring, 2004; Zhang et al., 2017). Built-up areas, on the other hand, have a major role in generating heat fluxes in urban dynamics. Water bodies naturally have a surface evaporation process as a result of solar radiation, which is why air moisture can control or decrease the air temperature of that particular area. Therefore, it could indicate that the land use/cover changes (LULCC) do have an intense effect on the surface radiant temperature of a location and it supports the phenomenon that anthropogenic land-use change is the vital reason leading to increases in LST in the urban micro-atmosphere (Jiang & Tian, 2010; Morabito et al., 2016).

Our study showed that expansion in developed areas had a significant effect on the LST but a nonsignificant effect on the green space, which is expected to be due to the upward expansion of buildings along with green areas, thus mitigating the effects of vegetation on LST. The temperature values found in densely vegetated/forest areas were low, while the highest values of LST were observed in an urban or built-up area of impervious surface compared with these two land covers of Chaoyang District, Beijing (Cai, Du & Xue, 2011; He et al., 2010; Yang, Ren & Liu, 2013) The plausible reasoning behind this fact is that increase in impermeable, hard and dark surfaces such as stone, metal, asphalt, and concrete building materials, increase land surface temperature by low reflection and high absorption of solar radiation, and emits heat not only during daytime but also at night time (Buyadi, Mohd & Misni, 2013; Oluseyi, Fanan & Magaji, 2009).

The available literature has shown that land cover classification could help in estimating the relationship between LST and various land-use types (Connors, Galletti & Chow, 2013; Walawender et al., 2014). UHI could be the result of obvious reasons, such as macro/mesoscale climate, urban morphology, population density, geographic location, anthropogenic changes in biophysical features of the surface area/land use, wind corridor, population pressures and human lifestyle, as well as the energy cycle. We observed that the trend in LULCC during the period 1990 to 2004 was quite large. That massive change in LULCC could be attributed to the rapid development of that area during that period. This period is considered to be one of intense deforestation and demolition of cropland for various developmental projects and shifted agricultural land to the evident level of barren land and built-up areas for housing and industry, characterized by a concomitant decline in total vegetation cover (VC) (Cai, Du & Xue, 2011; Chen et al., 2017). This rapid depletion of VC has a wide range of impacts on the reduction of natural cooling effects because of the shading and evapotranspiration of plants and shrubs (Macarof & Statescu, 2017; Oluseyi, Fanan & Magaji, 2009). To buttress this fact, (Weng, Lu & Schubring, 2004) the negative linear relationship between NDVI and LST demonstrated that VC acts as a sink within an UHI because of its cooling effects in an urban area. This change could eventually obliterate the surface evaporation and transpiration processes that mainly occur in plants (Faqe Ibrahim, 2017). Previous studies endorse this phenomenon that grasslands and ornamental plants have less impact on the reduction of LST than vegetation cover, such as forest/urban treebanks and gardens. (Chen et al., 2014; Fan et al., 2010; Weng & Lo, 2001).

Our results elucidate that land cover has a very dominant impact on LST in the urban environment. The impact values of green space, developed area and water bodies on their LST is according to their proportional area (Verburg et al., 2004; Xiong et al., 2012). This shows that vegetation plays an important role in mitigating or controlling the temperature in urban areas (Weng, Lu & Schubring, 2004). Through evaporation from the surface of the water bodies, moisture is added into the surrounding air. Build-up areas have a major role in generating heat fluxes in urban dynamics. Our results established a positive linear relationship between the LST and the built-up area (Chen & Zhang, 2017; Zhang & Su, 2016). The natural evaporation from the water body surfaces helps to cool down the surrounding air, thus decreasing the temperature of that area. Previous research also reported the impact of water bodies in the suburban areas apart from a significant role in controlling the LST (Chen & Zhang, 2017; Luo & Li, 2014)

These results established the phenomenon that urban sprawl is the primary factor in land surface temperature, which produces an abnormal heat flux in urban dynamics. This exchange of radiation/heat is considered a significant factor for the resulting UHI that contributes significantly to climate change in city canyons (Fig. 12). Therefore, LULCC does have an intense effect on the surface radiant temperature of a location, and it also seconds the phenomenon that anthropogenic LULCC is a main reason leading to an increase in LST in the urban micro-atmosphere. (Jiang & Tian, 2010)

### Impact and mitigation strategies of UHIs in urban dynamics

An urban heat island (UHI) diurnally varies with time of day, typically being most extensive in the morning. It also varies across different seasons. It depends on the synoptic conditions. The influential factors on the UHI are albedo, e.g., the shortwave solar radiation falls on the darker surfaces, deeper canyons and reflects back or is absorbed in a specific amount into various materials/surfaces (Grover & Singh, 2015; Saputra & Lee, 2019). Another contributor to UHIs is latent heat; cities loaded with large amounts of impervious surfaces, concrete, asphalt, steel and other hard-dark construction materials that do not allow moisture seepage into the soil for later evaporation which tend to cool the surface rather than storm sources (Deng et al., 2018).

Urban geometry plays an important role in trapping radiation, and the height and width of the urban canyon decide how much radiation can ultimately be trapped (Taha, Sailor & Akbari, 1992; Yue et al., 2019). Therefore, the impacts of thermal mass and radiation trapping on UHIs can be better catered by an effective “albedo modification strategy”, such as replacing low albedo material with high albedo material, which can be very effective in lowering the temperature of the absorbed surface and its surroundings (Taha, Sailor & Akbari, 1992; Yue et al., 2019). Moreover, a small modification of the engineering structure could also be helpful. The increasing population of Beijing requires more construction for housing, hospitals, schools and industries (Buckley & Simet, 2016). In the case of structural development, we should use material that not only reduces the impacts of UHIs but also shifts to CIs (cool islands). We contrived after analyzing the seasonal breeze/wind direction on the city master plans that building structure could be effective in cooling the air and providing protection from dust storm.

The second suggestion is the evaporative cooling in the urban canyons, which can be enhanced by effectively implementing the “green city project”, i.e., improve more green spaces by planting shade trees, shrubs and grasses in open places. However, this urban vegetation strategy mainly focuses on vertical vegetation on walls, roof gardening, surrounding green belts, vegetation on road banks and streets, ultimately enhancing evaporative cooling (Li et al., 2017; Tali et al., 2012; Zhang et al., 2017). Although the proportion of this green city project is very small compared to the infrastructure and population pressure, it can be a step forward, as many studies have confirmed that green infrastructure can mitigate the UHI effect up to 2−8 °C by enhancing the cooler urban breeze in very hot summers (Akbari et al., 1988; Lima Alves & Lopes, 2017; Taha, Sailor & Akbari, 1992).

Anthropogenic heating is a key influential factor that contributes to the increase in greenhouse gases, air pollution, urban heat fluxes, etc, which collaboratively enhanced the effect of the UHI for the last ten years. Anthropogenic heating is waste heat emission from transportation, residential and commercial buildings, industrial operations and urban development. The third strategy might be to improve open places in the city, e.g., the concept of open cinema/theatre, parks, open markets, might be a more effective strategy to conserve energy as well as mitigate the greenhouse gases by the use of indoor facilities such as air conditioners, fridges and other machinery, which might be less necessary to run this system (McCarthy et al., 2001). Anthropogenic heating is a key influential factor that contributes to the increase in greenhouse gases, air pollution, and urban heat fluxes, which collectively enhance the effect of urban heat islands (Lima Alves & Lopes, 2017; Weng, Lu & Schubring, 2004). This is the waste heat emission of transportation, residential and commercial buildings, industrial operations and urban development. This strategy enhances the collective wisdom and sense of responsibility to maintain an eco-friendly society along with all discussed mitigation options.

Finally, an urban heat mitigation strategy should be unique in its kind by which we can achieve maximum social, economic and environmental benefits. Although the government is playing and will continue to play a vital role in the mitigation of UHI effects, it is above that. To better face and manage the extremes of this major problem help of “integrated UHI mitigation efforts” from all stakeholders, including society, industry and government is needed. Everyone must act as a responsible member for the development of a healthy eco-friendly society. Moreover, capacity building in society needs to be enhanced by the collective efforts of the government, education sector and media.

## Conclusions

The present study assessed the LULCC effect on LST in the urban dynamics of Chaoyang using RS data in conjunction with observations of developmental revolution and various socioeconomic parameters. This study established the connectivity between the LST and various land covers. The contribution of landscape composition and its impacts on temperature were assessed by the determination of coefficient analysis and forecasting its immediate impact by using the Markov model. An increase in every 5% built-up area caused a 1% increase in temperature, and an increment of 10% in vegetation cover was also negatively correlated. This study concluded that the increase in land surface temperature (LST) was 5.5 °C, which was approximately 10.35% of the overall rise throughout the study period (2018–2025), showing its remarkable contribution to heat intensity in urban dynamics. The area of focus must be on urban design and infrastructure planning and development. The enhancement of water bodies such as lakes, canals, waterfall, and fountains, and considerable increases in green spaces, such as artificial parks, gardens and linear plantations, especially woody plants, may have certain positive impacts on mitigation activity. Alteration processes should be limited, and environmental education should be reawakened to accomplish the desired ecological development concerning environmental resource planning and management. The present study provides useful implications for urban landscape planning that require rational use of landscape connectivity between green and impervious surfaces and their impact on LST. Future urban research could focus on the issue of public health and infrastructure burden associated with rapid urbanization.

##  Supplemental Information

10.7717/peerj.9115/supp-1 Supplemental Information 1 Correlation anlysis results by R-studio

10.7717/peerj.9115/supp-2 Supplemental Information 2 Script for Correlation analysis and visualization in R-studio

10.7717/peerj.9115/supp-3 Supplemental Information 3 Script for Markove chain models for simulation analysis

## Additional Information and Declarations

### Data Availability

The following information was supplied regarding data availability: Data is available at FigShare: Liu, Dongyun (2019): Assessment and simulation of land use and land cover change impacts on the land surface temperature of Chaoyang District in Beijing, China.. figshare. Dataset. https://doi.org/10.6084/m9.figshare.9943571.v2.
